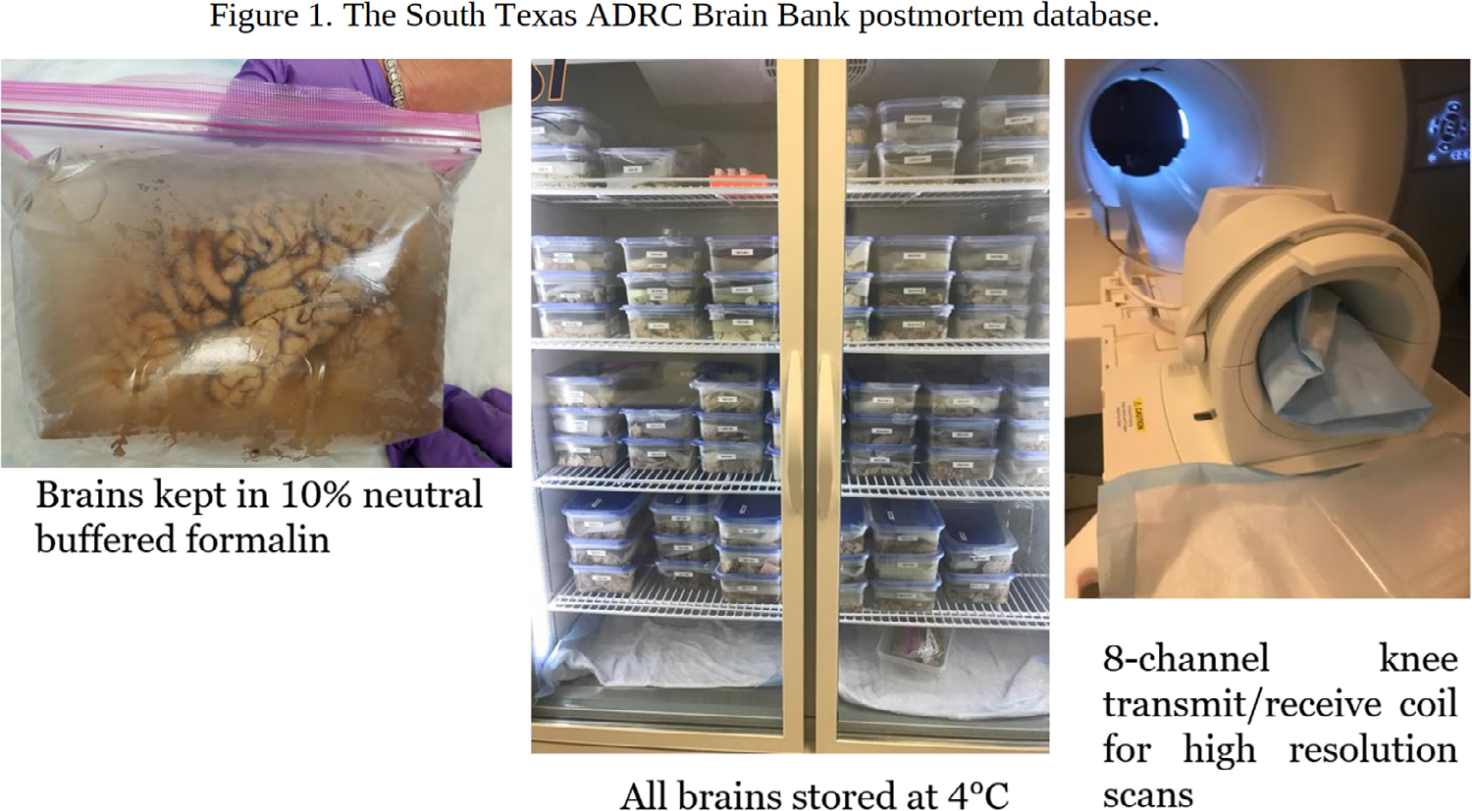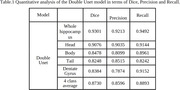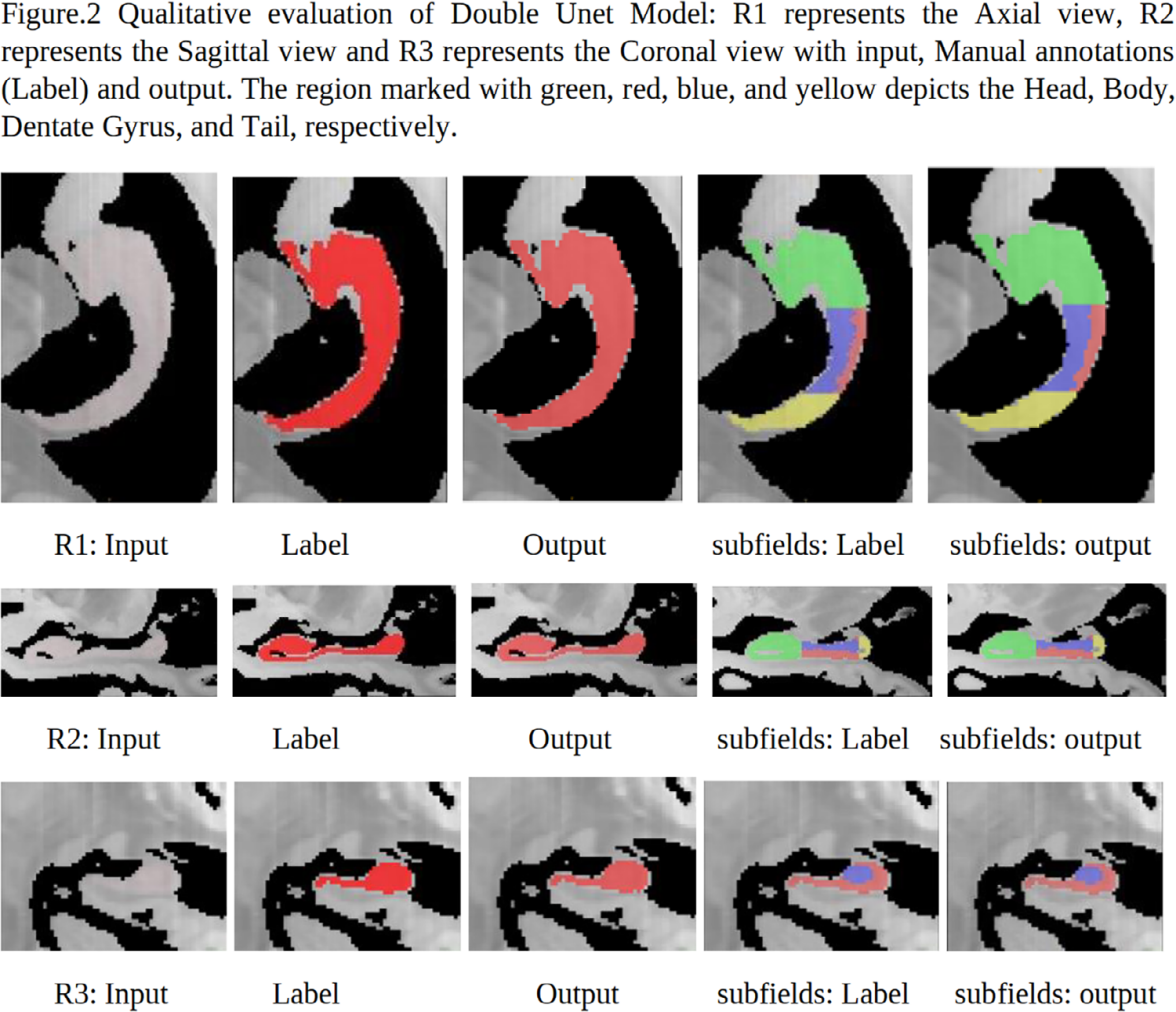# Deep Learning based Automated Segmentation of Hippocampus and Hippocampal Subfields from Postmortem MRI

**DOI:** 10.1002/alz.087748

**Published:** 2025-01-09

**Authors:** Anoop Benet Nirmala, Karl Li, Tanweer Rashid, Nicolas Honnorat, Di Wang, Jinqi Li, Elyas Fadaee, Sokratis Charisis, Jamie M. Walker, Timothy E. Richardson, David A Wolk, Peter T Fox, Kevin F. Bieniek, Sudha Seshadri, Laura E.M. Wisse, Mohamad Habes

**Affiliations:** ^1^ Glenn Biggs Institute for Alzheimer’s & Neurodegenerative Diseases, University of Texas Health Sciences Center at San Antonio, San Antonio, TX USA; ^2^ University of Texas Health San Antonio, San Antonio, TX USA; ^3^ Neuroimage Analytics Laboratory (NAL) and the Biggs Institute Neuroimaging Core (BINC), Glenn Biggs Institute for Alzheimer’s & Neurodegenerative Diseases, University of Texas Health Sciences Center, San Antonio, TX USA; ^4^ UT Health San Antonio, San Antonio, TX USA; ^5^ Research Imaging Institute, University of Texas Health Science Center at San Antonio, San Antonio, TX USA; ^6^ Neuroimage Analytics Laboratory and Biggs Institute Neuroimaging Core, Glenn Biggs Institute for Neurodegenerative Diseases, University of Texas Health Science Center at San Antonio, San Antonio, TX USA; ^7^ Icahn School of Medicine at Mount Sinai, New York, NY USA; ^8^ Department of Pathology, Icahn School of Medicine at Mount Sinai, New York, NY USA; ^9^ Department of Neurology, University of Pennsylvania, Philadelphia, PA USA; ^10^ Department of Clinical Sciences Lund, Lund University, Lund, Lund Sweden

## Abstract

**Background:**

The hippocampus and its subfields in the human brain play a pivotal role in forming new memories and spatial navigation. The automated assessment of the hippocampus and its subfields are useful tools for the early diagnosis of Alzheimer's disease and other neurodegenerative diseases such as primary age‐related tauopathy, Lewy body dementia, limbic‐predominant age‐related TDP‐43 encephalopathy (LATE), and frontotemporal lobar Dementia. Postmortem brain magnetic resonance imaging plays a crucial role in neuroscience and clinical research, providing valuable insights into the structural and pathological features of the brain after death. Postmortem MRI can be used to validate findings from in vivo imaging studies, link these findings to histopathological data. Deep learning methods such as convolutional neural networks are widely used in image segmentation. However, deep learning methods have not previously been applied to segmenting the hippocampus and its subfields (Head, Body, Tail, and Dentate Gyrus) from postmortem MRI.

**Method:**

Double U‐Net is a convolutional neural network architecture that combines two U‐Net architectures. The U‐Net architecture is widely used for semantic segmentation tasks, particularly in medical image analysis, where it has shown success in functions such as tumor segmentation. We have trained a double U‐Net model on 15 annotated postmortem MRI scans include scans from nine females and six males, with an average age of 78 and a standard deviation of 7, which have been collected at our university brain bank (Figure 1). Before feeding the data to the model, all the data were registered, intensity normalized, and cropped to a size of 64 x 96 x 48. The model is trained with a learning rate of 0.0001s and dice loss as the objective function.

**Result:**

The model's performance is evaluated quantitatively (Table 1) and qualitatively (Figure 2). The qualitative analysis is performed with metrics such as Dice, Precision, and Recall. In whole hippocampus segmentation, the model achieved an overall Dice score of 93.01%, and for subfield segmentation, it reached a Dice score of 87.30%.

**Conclusion:**

Deep learning‐based segmentation of the hippocampus and its subfields can be performed on postmortem brain images and may be helpful in diagnosing Alzheimer's and other neurodegenerative diseases.